# Pregnant women are a reservoir of malaria transmission in Blantyre, Malawi

**DOI:** 10.1186/1475-2875-13-506

**Published:** 2014-12-17

**Authors:** Sarah Boudová, Lauren M Cohee, Linda Kalilani-Phiri, Phillip C Thesing, Steve Kamiza, Atis Muehlenbachs, Terrie E Taylor, Miriam K Laufer

**Affiliations:** Center for Vaccine Development, School of Medicine, University of Maryland, Baltimore, MD USA; University of Malawi College of Medicine, Blantyre, Malawi; Blantyre Malaria Project, University of Malawi College of Medicine, Blantyre, Malawi; Department of Histopathology, University of Malawi College of Medicine, Blantyre, Malawi; Department of Pathology, University of Washington, Seattle, WA USA; Department of Osteopathic Medical Specialties, College of Osteopathic Medicine, Michigan State University, East Lansing, MI USA

**Keywords:** Malaria, Pregnancy, Malawi, Gametocyte, Intermittent preventive treatment, Sulphadoxine-pyrimethamine

## Abstract

**Background:**

During pregnancy, women living in malaria-endemic regions are at increased risk of malaria infection and can harbour chronic placental infections. Intermittent preventive treatment with sulphadoxine-pyrimethamine (SP-IPTp) is administered to reduce malaria morbidity. It was hypothesized that the presence of placental malaria infection and SP-IPTp use would increase the risk of peripheral blood gametocytes, the parasite stage that is transmissible to mosquitoes. This would suggest that pregnant women may be important reservoirs of malaria transmission.

**Methods:**

Light microscopy was used to assess peripheral gametocytaemia in pregnant women enrolled in a longitudinal, observational study in Blantyre, Malawi to determine the association between placental malaria and maternal gametocytaemia. The relationship between SP-IPTp and gametocytaemia was also examined.

**Results:**

2,719 samples from 448 women were analysed and 32 episodes of microscopic gametocytaemia were detected in 27 women. At the time of enrolment 22 of 446 women (4.9%) had gametocytaemia and of the 341 women for whom there was sufficient sampling to analyse infection over the entire course of pregnancy, 27 (7.9%) were gametocytaemic at least once. Gametocytaemia at enrolment was associated with placental malaria, defined as malaria pigment or parasites detected by histology or qPCR, respectively (OR: 32.4, 95% CI: 4.2-250.2), but was not associated with adverse maternal or foetal outcomes. Administration of SP-IPTp did not affect gametocyte clearance or release into peripheral blood.

**Conclusions:**

Gametocytaemia is present in 5% of pregnant women at their first antenatal visit and associated with placental malaria. SP-IPTp does not alter the risk of gametocytaemia. These data suggest that pregnant women are a significant reservoir of gametocyte transmission and should not be overlooked in elimination efforts. Interventions targeting this population would benefit from reaching women prior to first antenatal visit.

## Background

Malaria is a major cause of morbidity and mortality worldwide and it disproportionately affects children and pregnant women. Every year, 125 million pregnancies occur in malaria-endemic countries [1]. Pregnancy-associated malaria can result in maternal anaemia, low birth weight, prematurity, and increased infant mortality [2–8]. Women become vulnerable to malaria during their first and second pregnancies because they lack adequate immunity to variant surface proteins expressed by the parasite on the surface of infected red blood cells, allowing the infected cells to sequester in the placenta, a protected environment where the parasite can remain throughout pregnancy [9–12]. While asexual blood stage malaria infection causes malaria disease and is the focus of surveillance and prevention studies that have targeted pregnant women, sexual stage gametocyte infection is responsible for malaria transmission to the mosquito. Pregnant women have largely been overlooked as a potential reservoir for gametocytes despite their increased susceptibility to malaria, and prolonged infections due to placental sequestration. As interest in malaria elimination increases, the extent to which pregnant women with placental malaria contribute to the burden of malaria transmission requires investigation.

In addition to placental sequestration increasing the risk of malaria infection and gametocytaemia, anti-malarial interventions may impact the transmission potential of pregnant women. The current strategy to protect against the adverse effects of placental malaria is intermittent preventive treatment in pregnancy with sulphadoxine-pyrimethamine (SP-IPTp) given two to three times during pregnancy [13]. However, evidence suggests that SP can cause release of sequestered gametocytes into the peripheral blood that may persist at low levels for over a month [14–17]. Thus, the use of SP-IPTp may also increase the likelihood that pregnant women will have gametocytaemia.

To test the hypothesis that pregnant women serve as reservoirs of malaria transmission in malaria-endemic settings, the prevalence of gametocytaemia during pregnancy was measured in an observational study. The observational study reflected the natural history of gametocytaemia in pregnant women in a malaria-endemic area who received standard dosing of SP-IPTp. The association between placental infection or SP-IPTp and the presence of gametocytes was assessed.

## Methods

### Study site and population

Pregnant women were offered enrolment in an observational study of pregnant women conducted in Ndirande, a peri-urban township of Blantyre, Malawi. Malaria transmission occurs year-round with a seasonal peak during the three- to four-month rainy season. Women were enrolled at their first antenatal visit if they were in their first or second pregnancy and at less than or equal to 28 weeks of gestation. Women were excluded if they had any major illness requiring high-risk obstetric care, HIV infection or chronic antibiotic treatment. Women were followed through delivery. Details of the study design and primary study results have been published previously [18]. Gestational age at enrolment was calculated based on the last menstrual period or by fundal height if the last menstrual period was not known. Gestational age at delivery was estimated based on the last menstrual period and the Ballard score; the fundal height at enrolment was included in the estimate if the last menstrual period and Ballard estimates were more than two weeks discrepant.

### Study procedures

Women attended routine clinic visits every four weeks and were encouraged to return any time they were ill. At all visits, including routine visits and whenever there was an illness with symptoms suggestive of malaria, history and physical examination were performed, with axillary temperature recorded and a fever defined as ≥37.5°C. Thick and thin malaria smears were collected and drops of blood were collected on filter paper. Malaria smears were field-stained and examined using light microscopy with a 100× oil immersion lens. Parasitaemia was assessed by counting the number of parasites per 200 leukocytes and examining 100 high-power fields before considering a smear to be malaria-negative. All slides were read by two microscopists and in cases of disagreement between the readings, the conflict was adjudicated by a third expert reader. Parasite density was calculated based on 8,000 leukocytes per microlitre. All smears identified as having parasitaemia in the observational study were classified as having asexual-stage infection and were treated with artemether-lumefantrine.

Participants were given SP-IPTp up to three times in pregnancy during prenatal follow-up, in accordance with national policy in Malawi. At delivery the placenta was collected for molecular and histopathological examination. Newborns were assessed and gestational age was estimated as previously described [19]. Haemoglobin measurements were obtained from a finger-prick blood sample using HemoCue® AB. Ethical approval was obtained from the University of Malawi College of Medicine’s Research Ethics Committee and the University of Maryland Baltimore Institutional Review Board. Written informed consent was obtained from all participants before conducting any study-related activities. Participants had the option to withdraw from the study at any time. All data were recorded and analysed anonymously.

### Identification of gametocytes

After completion of the observational study, dried blood spots collected on filter paper from all antenatal visits were subjected to quantitative real-time PCR (qPCR) for *Plasmodium falciparum* lactate dehydrogenase to detect infection. Extraction and PCR protocols are described online [20]. A peripheral blood specimen was considered uninfected if the qPCR was negative. For all qPCR-positive filter papers, the corresponding blood smear was examined for gametocytes; in cases where no filter paper specimens were available, the blood smear was examined for gametocytes. Smears re-examined for gametocytes were counted per 1,000 leukocytes. Gametocyte density was calculated based on 8,000 leukocytes per microlitre. The entire smear was scanned before being considered negative for gametocytes. All slides were read for gametocytes by one microscopist.

### Identification of placental malaria

Placental tissue biopsies were processed as previously described [18] and examined for the presence of malaria haemozoin and parasites. Placental blood filter papers were subjected to qPCR as described above. Placental malaria was defined as evidence of haemozoin by histology or placental parasites by qPCR. If the histology and the qPCR were negative, the placenta was considered uninfected.

### Data analysis

Dried blood spots and corresponding blood smears were examined from all women at enrolment prior to the administration of SP-IPTp. To determine the prevalence of gametocytaemia during pregnancy, data were included from all women who had gametocytaemia at any time during pregnancy. Women were classified as not having gametocytaemia during pregnancy if they had specimens available for analysis from enrolment and at least two months of follow-up. Data from one placenta of twin gestations were included in analyses of placental malaria but excluded from analyses of birth outcomes. When placental results were discordant, only the positive placental result was included.

To determine the effect of SP on gametocyte clearance, all women who were considered malaria-negative in the observational study (and therefore not treated with artemether-lumefantrine) but subsequently found to be gametocyte-positive at enrolment were included. To examine the effect of SP on gametocyte release, women who were gametocyte-negative by microscopy but had sub-microscopic infection (malaria-positive by qPCR, but negative by microscopy in the observational study and gametocyte analysis) at the time of enrolment were evaluated. In both analyses, the proportion of women with gametocytaemia at their visit one month after enrolment was compared using Fisher’s exact test.

Data analysis was performed using STATA version 12.0 software (Stata Corp, College Station, TX, USA). Descriptive statistics were analysed using frequencies and percentages for categorical data and geometric means, means, medians, and 95% confidence intervals for continuous variables. 95% confidence intervals were calculated for all proportions and prevalence rates. To measure prenatal risk factors and outcomes associated with gametocytaemia, logistic regression was used to calculate odds ratios. For the multivariate model, any parameter in the univariate model associated with gametocytaemia with p ≤ 0.1 was considered. Maternal treatment for malaria prior to enrolment, asexual stage parasitaemia at enrolment, mean maternal haemoglobin at enrolment, haemozoin in the placenta, and parasites detected in the placenta by qPCR were included. All p-values are two-sided and statistical significance was set at p ≤ 0.05.

## Results

### Prevalence of gametocytaemia and placental malaria

A total of 2,719 blood smears from 448 women were available and read for gametocytes if the corresponding filter-paper blood spot was qPCR-positive for malaria. Thirty-two episodes of gametocytaemia in 27 women were detected. Twenty-two (68.7%) of the gametocyte episodes occurred at the enrolment visit and all episodes of gametocytaemia occurred within two months of enrolment. Five women had gametocytaemia twice during their participation in the study. The geometric mean gametocyte density among gametocyte-positive smears was 19.9 gametocytes per microlitre of blood (95% CI: 13.3-29.8).

At enrolment, 22 of 446 (4.9%) smears were positive for gametocytes. Enrolment smears from two women were damaged and could not be evaluated. The gametocyte prevalence was approximately ten-fold higher among those women with asexual-stage malaria detected by light microscopy compared to those without infection detected by light microscopy (12/60, 20.0% *vs* 10/386, 2.6%). Among the 341 participants who had sufficient data to evaluate the presence of any gametocytes during pregnancy, 27 (7.9%) had at least one episode of gametocytaemia. The proportion of women with gametocytaemia during pregnancy was higher among women who had asexual-stage malaria detected by light microscopy at least once during pregnancy, 18 of 65 (27.7%), as compared to nine of 276 (3.3%, p < 0.01) for those without asexual-stage infection detectable by microscopy.

Molecular data was available from 321 placentas and histology was available for 311. One-hundred and ten (34.3%) women had evidence of placental malaria either by histology, qPCR or both. Among the 311 women with both histology and qPCR data available, 33 (10.6%) had placental parasites alone, 38 (12.2%) had haemozoin alone, 33 (10.6%) had both parasites and haemozoin, and 207 women had neither. Of the ten placentas for which histology was unavailable, six (60%) had placental malaria by qPCR.

### Risk factors associated with gametocytaemia at enrolment

At enrolment, gametocytaemia was associated with placental malaria, lower maternal haemoglobin and the presence of asexual stage infection. Age, gravidity, gestational age, history of malaria treatment, maternal temperature at enrolment and bed net use were not associated with gametocyte infection (Table 1). No women with malaria infection had complaints of febrile illness at their enrolment visit and only 7 of 102 (6.9%) women with malaria infection had a fever at the time of their evaluation. No women with gametocytaemia had a measured fever. Placental malaria, with evidence of haemozoin and/or qPCR, was associated with 30-fold increased odds of gametocytaemia at enrolment (Table 1). In univariate analysis both haemozoin and qPCR detection of placental malaria were associated with gametocytaemia at enrolment. In multivariate analysis, haemozoin was associated with gametocytaemia at enrolment (OR: 10.3, 95% CI: 1.9-55.1). Molecular detection of placental malaria, lower maternal haemoglobin at enrolment, and asexual stage infection at enrolment were not associated with gametocytaemia at enrolment.

**Table 1:** **The relationship between gametocytaemia at enrolment and placental malaria**

	Univariate analysis					Multivariate		
	Gametocytes	No gametocytes	OR	95% CI	P value	OR	95% CI	P value
Placental malaria (haemozoin or qPCR)	14/15 (93.3%)	89/295 (30.2%)	32.4	4.2 - 250.2	<0.01	--	--	--
Haemozoin	12/15 (80.0%)	58/295 (19.7%)	16.3	4.5-59.8	<0.01	10.3	1.9 – 55.1	<0.01
qPCR	9/15 (60.0%)	62/304 (20.4%)	5.9	2.0-17.1	<0.01	2.8	0.8 – 9.3	0.09
Mean maternal haemoglobin (g/dL) at enrolment (standard deviation)	10.9 (1.5)	11.8 (1.6)	0.7	0.6 – 1.0	0.02	1.1	0.7 – 1.5	0.76
Asexual stage parasitaemia at enrolment	12/22 (54.5%)	48/424 (11.3%)	9.4	3.9 – 22.9	<0.01	1.4	0.4 – 5.8	0.62
Malaria treatment prior to enrolment	6/22 (27.3%)	56/422 (13.2%)	2.5	0.9 – 6.5	0.09	2.8	0.8 – 10.1	0.11
Mean maternal age in years (standard deviation)	20.3 (3.1)	20.1 (3.2)	1.0	0.9 – 1.2	0.77	--	--	--
Primigravid	13/22 (59.1%)	268/424 (63.2%)	1.2	0.5 – 2.8	0.70	--	--	--
Mean gestational age in weeks at the time of enrolment (standard deviation)	20.1 (4.0)	20.1 (4.4)	1.0	0.9 – 1.1	0.97	--	--	--
Mother slept under a bed net the previous night	9/22 (40.9%)	211/422 (50.0%)	1.4	0.6 – 3.5	0.40	--	--	--
Maternal axillary temperature (°C) at enrolment (standard deviation)	36.5 (0.4)	36.6 (0.4)	0.8	0.3 – 2.2	0.72	--	--	--

### Gametocytaemia and maternal and foetal outcomes

Gametocytaemia at enrolment or at any time during pregnancy was not associated with differences in gestational age at birth, birth weight or maternal haemoglobin concentration at delivery.

### The effect of SP on gametocytaemia

**Figure 1:**
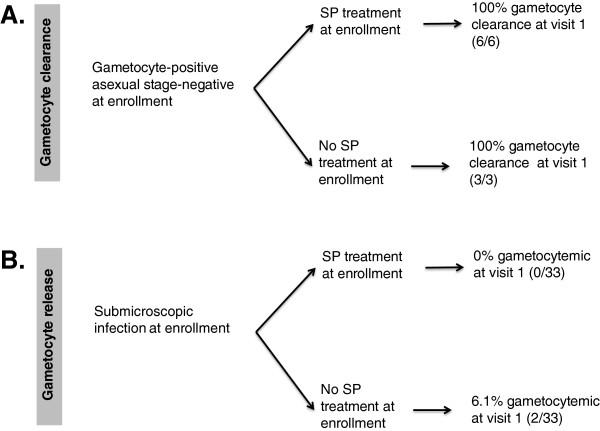
**The relationship between SP-IPTp and gametocytaemia. A**. Clearance of gametocyte infection within one month following treatment with SP vs no treatment. **B**. Release of gametocytes within one month after SP vs no treatment.

When examining women who had gametocytaemia at enrolment, all women cleared gametocytes within one month regardless of whether they received SP (Figure 1A). Among women with sub-microscopic infection, the rates of gametocytaemia one month later were low and were not different between women who did or did not receive SP-IPTp (0% *vs* 6.1%, p = 0.5, Figure 1B).

## Discussion

These data show 5% gametocyte prevalence at the time of first antenatal visit and increased odds of peripheral gametocytaemia among women with evidence of placental malaria at delivery. This association was independent of asexual stage parasitaemia. When examined separately in a multivariate model, the presence of haemozoin, was associated with gametocytaemia at the time of enrolment and the association with active placental malaria infection did not achieve statistical significance (p = 0.09). In the context of SP-IPTp and active case detection and treatment with artemether-lumefantrine, episodes of gametocytaemia were rare after initiating antenatal care. All episodes of gametocytaemia occurred within two months of enrolment. No association between SP and either gametocyte release into peripheral blood or gametocyte clearance was found.

This is the first study examining the relationship between placental malaria and gametocytaemia, and one of the few studies examining gametocytaemia in the context of pregnancy. A previous study in Tanzania documented a high prevalence of gametocytaemia (12-23%) in pregnant women with microscopically confirmed malaria [21]. This is comparable to the 20% gametocyte prevalence observed among women with asexual-smear-positive malaria at enrolment in this study. However, the Tanzanian study did not capture the full burden of gametocytaemia during pregnancy because only symptomatic women were included and most malaria infections during pregnancy are asymptomatic.

The prevalence of gametocytaemia among pregnant women in this study is similar to that of children in the region, suggesting that pregnant women serve as a potentially important reservoir of malaria transmission, just as malaria-naïve children do. The prevalence of gametocytes among children with symptomatic uncomplicated malaria was 22.6% in Tanzania [15], which is similar to the 20% found in this study. Gametocyte prevalence in the entire pregnant population at enrolment in this study was 4.9% consistent with the prevalence of 5.6% seen in a cross-sectional study of children in Mozambique [22].

A direct comparison cannot be made between pregnant and non-pregnant adults due to a lack of data on non-pregnant adult gametocyte prevalence in Malawi and neighbouring countries. However, it has been observed in multiple African settings, that with increasing age, gametocyte prevalence drops rapidly [23, 24].

Although the rates of gametocytaemia are similar between children and pregnant women, the transmission potential of pregnant women raises unique concerns. Their infections are almost always asymptomatic—as was the case for all women in this study at the time of enrolment—whereas children are more likely to exhibit symptoms of malaria leading to treatment intervention, potentially reducing their transmission potential. Although young children contribute a larger proportion of the transmission reservoir than pregnant women [25], the identification of pregnant women as an important source of transmission would require interventions that are different from young children.

There are several limitations to this characterization of gametocyte prevalence. All of the women enrolled were in their first or second pregnancy, and therefore at higher risk of pregnancy-associated malaria and placental malaria than multigravid women. Thus the gametocyte prevalence in the general pregnant population may be overestimated. However, because this study was limited to detecting gametocytes by light microscopy, the results likely underestimate the true prevalence of gametocytes. Light microscopy is insufficiently sensitive to detect all infections that are transmissible to mosquitoes. Comparing molecular methods with microscopy, Bousema *et al.* [15] observed over a three-fold increase in gametocyte prevalence. These sub-microscopic concentrations of gametocytes can be infectious to mosquitoes in a blood meal [26]. In addition, the circumstances of this clinical study likely led to an overall decrease in the observed prevalence of gametocytaemia. Women in the study were followed by active case detection and treated for all episodes of malaria infection with artemether-lumefantrine, even in the absence of symptoms. Women were only enrolled at their first antenatal visit, usually well into their second trimester, thus it is probable that cases of gametocytaemia were missed in the early pregnancy period.

The high rate of gametocytaemia and its association with placental malaria infection suggests that pregnancy, and possibly the presence of placental sequestration put pregnant women at risk for gametocytaemia. Gametocytes are very rarely found in the placenta [27], making sequestration of gametocytes themselves in the placenta an unlikely explanation for the association between placental malaria and peripheral gametocytaemia in pregnancy. While the observed association may reflect higher malaria exposure in women with gametocyte infection, there are several possible physiological explanations for this novel finding. First, gametocytes may express, pregnancy-specific surface proteins against which women lack immunity. This seems possible since asexual stages are known to express a novel surface protein, VAR2CSA during pregnancy, against which primi- and secondi-gravid women lack immunity [11, 12]. Another possibility is that adult women have weak gametocyte-specific immunity. Adults living in malaria-endemic regions develop protective immunity against asexual-stage parasites with repeated exposure. Efficient immune clearance of parasites may prevent parasites from surviving long enough to develop into gametocytes. In support of this theory, it has been demonstrated that gametocyte-specific immunity is inversely correlated with age [28]. However during pregnancy, asexual stage parasites evade immune clearance via placental sequestration and thus have the potential to develop into gametocytes. Since adults have weak gametocyte-specific immunity, the sexual stage of the parasite may persist resulting in an increased prevalence of gametocytaemia during pregnancy. Finally, pregnancy-specific changes to the bone marrow, the site of gametocyte enrichment and development [29], could alter gametocyte prevalence. Pregnancy causes bone marrow hyperplasia and reticulocytosis [30]. Structural changes to the bone marrow could reduce the extravascular space in the bone marrow forcing gametocytes into the periphery. Alternatively, bone marrow hyperplasia could result in an expansion of the environment where gametocytes develop and a subsequent increase in gametocyte production. Although the mechanism is unknown, reticulocytes have been shown *in vitro* to increase gametocytogenesis [31]. The increase in reticulocyte levels in pregnancy could drive gametocyte production and thus gametocyte prevalence. The mechanism by which pregnancy promotes gametocytaemia merits further examination.

In this limited sample size, no association between SP prophylaxis and gametocytaemia was detected. This is consistent with a previous study demonstrating that SP-IPT in children decreased gametocytaemia [32]. The increase in gametocytaemia typically occurs one week after SP administration [14, 16, 17], so the one-month sampling time frame may have missed a transient rise in gametocytes. However, the overall findings indicate that microscopically detectable gametocytaemia disappeared by three months of study interventions, which included repeated dosing of SP-IPTp, providing support for the conclusion that SP-IPTp does not promote gametocyte production or release.

Most cases of gametocytaemia in this study occurred at the time of enrolment, suggesting that pregnant women may be an important source of malaria transmission early in pregnancy. This has significant public health implications. It highlights the need for interventions aimed at preventing malaria to reach women earlier in pregnancy. This study and others [18, 19] have clearly demonstrated the need to reach pregnant women early in their pregnancy, before malaria infection occurs. This is particularly concerning since it is typical for women in sub-Saharan Africa to wait until half-way through pregnancy before seeking antenatal care [33, 34] potentially allowing gametocytaemia to persist for months. The results of this study highlight the urgent need to promote antenatal attendance early in pregnancy. In addition to expanding opportunities to clear gametocytaemia, other benefits include earlier access to sexually transmitted disease testing, iron-folate supplements and insecticide-treated nets. If cultural sensitivity about revealing pregnancy status early in gestation cannot be overcome [35], this study and others justify the exploration of interventions that target all women of child-bearing potential [18, 19]. There is an urgent need for alternatives to SP that can be used early in pregnancy, are not compromised by resistance and are different enough from current first-line therapy to avoid selection for resistant parasites. In Malawi and other areas of Africa, the return of chloroquine-susceptible malaria after cessation of chloroquine use raises the possibility that chloroquine alone or in combination may serve this purpose. Studies are currently underway to evaluate this option.

It is also possible that maternal gametocytaemia could impact foetal and infant immunity. Gametocytaemia could result in increased production of gametocyte-specific antibodies that would be transferred to the infant providing protection against gametocyte carriage in infancy. Like asexual-stage proteins [36], gametocyte proteins could traverse the placenta in immune complexes causing foetal immune priming with the potential to predispose the infant to either elevated effector responses to gametocytes or immune tolerance to this life stage.

The results of this study raise the possibility that activity against gametocytes may be important in the selection of strategies to prevent and control pregnancy-associated malaria. Although all cases of gametocytaemia were cleared within three months of participation in the study, study interventions were extensive, including SP-IPTp, which is not gametocidal, and treatment with artemether-lumefantrine, which is known to have some activity against gametocytes. This study was not able to evaluate the relationship between artemether-lumefantrine treatment and gametocytaemia. It is possible that clearance of asexual stages via IPTp is sufficient to prevent gametocyte development from the asexual stages, and thus prevent gametocytaemia. Alternatively, the treatment targeting asexual stages may allow gametocytes to remain in the periphery and bone marrow, or may even promote gametocytogenesis. IPTp with drugs with known gametocidal activity, such as the artemisinin derivatives may be necessary to prevent parasite transmission. Further study is needed to elucidate the mechanism of gametocytogenesis and determine which drugs are the most effective in clearing infections and also preventing transmission. The results of this study highlight the critical role of preventing malaria in pregnant women, not only to protect the health of the mother and the infant but also to make progress towards malaria elimination.

## Conclusion

Gametocytaemia was observed in 5% of pregnant women before they initiated antenatal care and began receiving IPTp. Placental malaria is associated with increased odds of gametocytaemia in pregnancy independently of asexual stage parasitaemia. In this study, there is no evidence that SP-IPTp increases the presence of gametocytes. Overall these data suggest that pregnant women may be a significant reservoir of gametocytes and should not be overlooked in malaria elimination strategies.

## Abbreviations

IPTp: Intermittent preventive treatment in pregnancy
qPCR: Quantitative polymerase chain reaction
SP: Sulphadoxine-pyrimethamine.

## References

[1] Dellicour S, Tatem AJ, Guerra CA, Snow RW, Ter Kuile FO (2010). Quantifying the number of pregnancies at risk of malaria in 2007: a demographic study. PLoS Med.

[2] Rogerson SJ, Chaluluka E, Kanjala M, Mkundika P, Mhango C, Molyneux ME (2000). Intermittent sulfadoxine-pyrimethamine in pregnancy: effectiveness against malaria morbidity in Blantyre, Malawi, in 1997–99. Trans R Soc Trop Med Hyg.

[3] Abrams ET, Kwiek JJ, Mwapasa V, Kamwendo DD, Tadesse E, Lema VM, Molyneux ME, Rogerson SJ, Meshnick SR (2005). Malaria during pregnancy and foetal haematological status in Blantyre Malawi. Malar J.

[4] Steketee RW, Wirima JJ, Hightower AW, Slutsker L, Heymann DL, Breman JG (1996). The effect of malaria and malaria prevention in pregnancy on offspring birthweight, prematurity, and intrauterine growth retardation in rural Malawi. Am J Trop Med Hyg.

[5] Sullivan AD, Nyirenda T, Cullinan T, Taylor T, Harlow SD, James SA, Meshnick SR (1999). Malaria infection during pregnancy: intrauterine growth retardation and preterm delivery in Malawi. J Infect Dis.

[6] Kalanda BF, van BS, Verhoeff FH, Brabin BJ (2005). Anthropometry of Malawian live births between 35 and 41 weeks of gestation. Ann Hum Biol.

[7] Verhoeff FH, Brabin BJ, Chimsuku L, Kazembe P, Broadhead RL (1999). Malaria in pregnancy and its consequences for the infant in rural Malawi. Ann Trop Med Parasitol.

[8] Hartman TK, Rogerson SJ, Fischer PR (2010). The impact of maternal malaria on newborns. Ann Trop Paediatr.

[9] Rogerson SJ, Hviid L, Duffy PE, Leke RF, Taylor DW (2007). Malaria in pregnancy: pathogenesis and immunity. Lancet Infect Dis.

[10] Fried M, Duffy PE (1996). Adherence of *Plasmodium falciparum* to chondroitin sulfate A in the human placenta. Science.

[11] Salanti A, Staalsoe T, Lavstsen T, Jensen AT, Sowa MP, Arnot DE, Hviid L, Theander TG (2003). Selective upregulation of a single distinctly structured var gene in chondroitin sulphate A-adhering *Plasmodium falciparum* involved in pregnancy-associated malaria. Mol Microbiol.

[12] Duffy MF, Caragounis A, Noviyanti R, Kyriacou HM, Choong EK, Boysen K, Healer J, Rowe JA, Molyneux ME, Brown GV, Rogerson SJ (2006). Transcribed var genes associated with placental malaria in Malawian women. Infect Immun.

[13] World Health Organization (2004). Strategic Framework for Malaria Prevention and Control During Pregnancy in the African Region.

[14] Bousema JT, Schneider P, Gouagna LC, Drakeley CJ, Tostmann A, Houben R, Githure JI, Ord R, Sutherland CJ, Omar SA, Sauerwein RW (2006). Moderate effect of artemisinin-based combination therapy on transmission of *Plasmodium falciparum*. J Infect Dis.

[15] Bousema T, Okell L, Shekalaghe S, Griffin JT, Omar S, Sawa P, Sutherland C, Sauerwein R, Ghani AC, Drakeley C (2010). Revisiting the circulation time of *Plasmodium falciparum* gametocytes: molecular detection methods to estimate the duration of gametocyte carriage and the effect of gametocytocidal drugs. Malar J.

[16] von SL, Drakeley C, Greenwood B, Walraven G, Targett G (2001). Risk factors for gametocyte carriage in Gambian children. Am J Trop Med Hyg.

[17] Targett G, Drakeley C, Jawara M, von SL, Coleman R, Deen J, Pinder M, Doherty T, Sutherland C, Walraven G, Milligan P (2001). Artesunate reduces but does not prevent posttreatment transmission of *Plasmodium falciparum* to *Anopheles gambiae*. J Infect Dis.

[18] Kalilani-Phiri L, Thesing PC, Nyirenda OM, Mawindo P, Madanitsa M, Membe G, Wylie B, Masonbrink A, Makwakwa K, Kamiza S, Muehlenbachs A, Taylor TE, Laufer MK (2013). Timing of malaria infection during pregnancy has characteristic maternal, infant and placental outcomes. PLoS One.

[19] Cohee LM, Kalilani-Phiri L, Boudova S, Joshi S, Mukadam R, Seydel KB, Mawindo P, Thesing P, Kamiza S, Makwakwa K, Muehlenbachs A, Taylor TE, Laufer MK (2014). Submicroscopic malaria infection during pregnancy and the impact of intermittent preventive treatment. Malar J.

[20] University of Maryland Center for Vaccine Development (2014). Malaria Group Protocols.

[21] Mutabingwa TK, Muze K, Ord R, Briceno M, Greenwood BM, Drakeley C, Whitty CJ (2009). Randomized trial of artesunate + amodiaquine, sulfadoxine-pyrimethamine + amodiaquine, chlorproguanal-dapsone and SP for malaria in pregnancy in Tanzania. PLoS One.

[22] Mabunda S, Casimiro S, Quinto L, Alonso P (2008). A country-wide malaria survey in Mozambique I. Plasmodium falciparum infection in children in different epidemiological settings. Malar J.

[23] Ouedraogo AL, Bousema T, de Vlas SJ, Cuzin-Ouattara N, Verhave JP, Drakeley C, Luty AJ, Sauerwein R (2010). The plasticity of *Plasmodium falciparum* gametocytaemia in relation to age in Burkina Faso. Malar J.

[24] Drakeley C, Sutherland C, Bousema JT, Sauerwein RW, Targett GA (2006). The epidemiology of *Plasmodium falciparum* gametocytes: weapons of mass dispersion. Trends Parasitol.

[25] WHO (2013). World Health Statistics 2013.

[26] El-Sayed B, El-Zaki SE, Babikr H, Gadalla N, Ageep T, Mansour F, Baraka O, Babiker A (2007). A randomized open-label trial of artesunate-sulfadoxine-pyrimethamine with or without primaquine for elimination of sub-microscopic *P. falciparum* parasitemia and gametocyte carriage in eastern Sudan. Plos One.

[27] Desowitz RS, Buchbinder G (1992). The absence of *Plasmodium falciparum* gametocytes in the placental blood of a woman with a peripheral parasitaemia. Ann Trop Med Parasitol.

[28] Drakeley CJ, Bousema JT, Akim NI, Teelen K, Roeffen W, Lensen AH, Bolmer M, Eling W, Sauerwein RW (2006). Transmission-reducing immunity is inversely related to age in *Plasmodium falciparum* gametocyte carriers. Parasite Immunol.

[29] Joice R, Nilsson SK, Montgomery J, Dankwa S, Egan E, Morahan B, Seydel KB, Bertuccini L, Alano P, Williamson KC, Duraisingh MT, Taylor TE, Milner DA, Marti M (2014). *Plasmodium falciparum* transmission stages accumulate in the human bone marrow. Sci Transl Med.

[30] Lowenstein L, Bramlage CA (1957). The bone marrow in pregnancy and the puerperium. Blood.

[31] Trager W, Gill GS, Lawrence C, Nagel RL (1999). *Plasmodium falciparum*: enhanced gametocyte formation *in vitro* in reticulocyte-rich blood. Exp Parasitol.

[32] Schellenberg D, Menendez C, Aponte JJ, Kahigwa E, Tanner M, Mshinda H, Alonso P (2005). Intermittent preventive antimalarial treatment for Tanzanian infants: follow-up to age 2 years of a randomised, placebo-controlled trial. Lancet.

[33] Crawley J, Hill J, Yartey J, Robalo M, Serufilira A, Ba-Nguz A, Roman E, Palmer A, Asamoa K, Steketee R (2007). From evidence to action? Challenges to policy change and programme delivery for malaria in pregnancy. Lancet Infect Dis.

[34] Abou-Zahr CL, Warhurst D (2003). Antenatal care in developing countries: promises, achievements and missed opportunities: an analysis of trends, levels and differentials.

[35] Brighton A, D’Arcy R, Kirtley S, Kennedy S (2013). Perceptions of prenatal and obstetric care in Sub-Saharan Africa. Int J Gynaecol Obstet.

[36] May K, Grube M, Malhotra I, Long CA, Singh S, Mandaliya K, Siegmund W, Fusch C, Schneider H, King CL (2009). Antibody-dependent transplacental transfer of malaria blood-stage antigen using a human *ex vivo* placental perfusion model. PLoS One.

